# Laparoscopic repair of a primary parahiatal hernia combined with gastric volvulus: a case report and literature review

**DOI:** 10.1186/s40792-024-01931-9

**Published:** 2024-05-31

**Authors:** Hirotada Muramatsu, Hisashi Amaike, Rena Ogura, Kouichi Shirono, Noriyuki Kamiya

**Affiliations:** 1 Department of Surgery, Ito Municipal Hospital, 196-1 Oka, Ito, Shizuoka Japan; 2 Department of General Surgery, Tokyo Bay Urayasu Ichikawa Medical Center, 3-4-32 Toudaijima, Urayasu, Chiba Japan

**Keywords:** Parahiatal hernia, Laparoscopic surgery, Fundoplication

## Abstract

**Background:**

Parahiatal hernias present a hernial orifice at the diaphragm that is adjacent to the esophageal hiatus, differing from the paraesophageal type of hiatal hernias. Although diagnostic imaging has advanced in recent years, diagnosing parahiatal hernias remains challenging. We herein report a case in which we performed laparoscopic surgery and intraoperatively diagnosed a parahiatal hernia.

**Case presentation:**

A 67-year-old man presented to our hospital with difficulty eating, epigastric pain, and vomiting. We suspected a paraesophageal hiatal hernia. Laparoscopic surgery was performed, and a diagnosis of parahiatal hernia was made. We closed the hernial orifice with direct simple closure using nonabsorbable threads. The patient’s postoperative recovery course was reasonable, and he was discharged on the twelfth postoperative day.

**Conclusions:**

Parahiatal hernias are rare, and a definitive diagnosis is difficult. Laparoscopic surgery can help accurately diagnose and treat patients presenting with the condition.

## Background

Hiatal hernias are the most common diaphragmatic hernias in adults. Most hiatal hernias are of the sliding type, in which the esophagogastric (EG) junction is located in the thoracic cavity. Paraesophageal hiatal hernias at the EG junction in the abdominal cavity are relatively rare. In contrast, a parahiatal hernia has a hernial orifice at the diaphragm, adjacent to the esophageal hiatus. Though reports of parahiatal hernias are increasing, they still remain rare. Patients with parahiatal hernias present symptoms similar to those of a paraesophageal hiatal hernia, such as difficulty in eating. Despite the advances in diagnostic imaging, diagnosing parahiatal hernias remains challenging. The diagnosis is established by confirming the presence of diaphragmatic tissue between the hernial orifice and the esophageal hiatus. Herein, we report the case of a parahiatal hernia presenting with severe epigastralgia and dysphagia. We employed computed tomography (CT) and preoperatively suspected a paraesophageal hiatal hernia with gastric volvulus. However, a parahiatal hernia was diagnosed in view of the intraoperative findings during laparoscopic surgery.

## Case presentation

A 67-year-old man presented with long-term occasional heartburn, without any previous examination or treatments. A week before his visit, he started noticing difficulty eating, postprandial epigastric pain, and vomiting. His symptoms persisted, and he consulted his physician. The patient had no history of trauma. Chest radiography showed air in the left thoracic cavity; therefore, a hiatal hernia was suspected. Esophagogastroduodenoscopy (EGD) showed outflow stenosis. Therefore, he was diagnosed with a hiatal hernia with gastric volvulus and was transferred to our hospital for treatment.

**Fig. 1:**
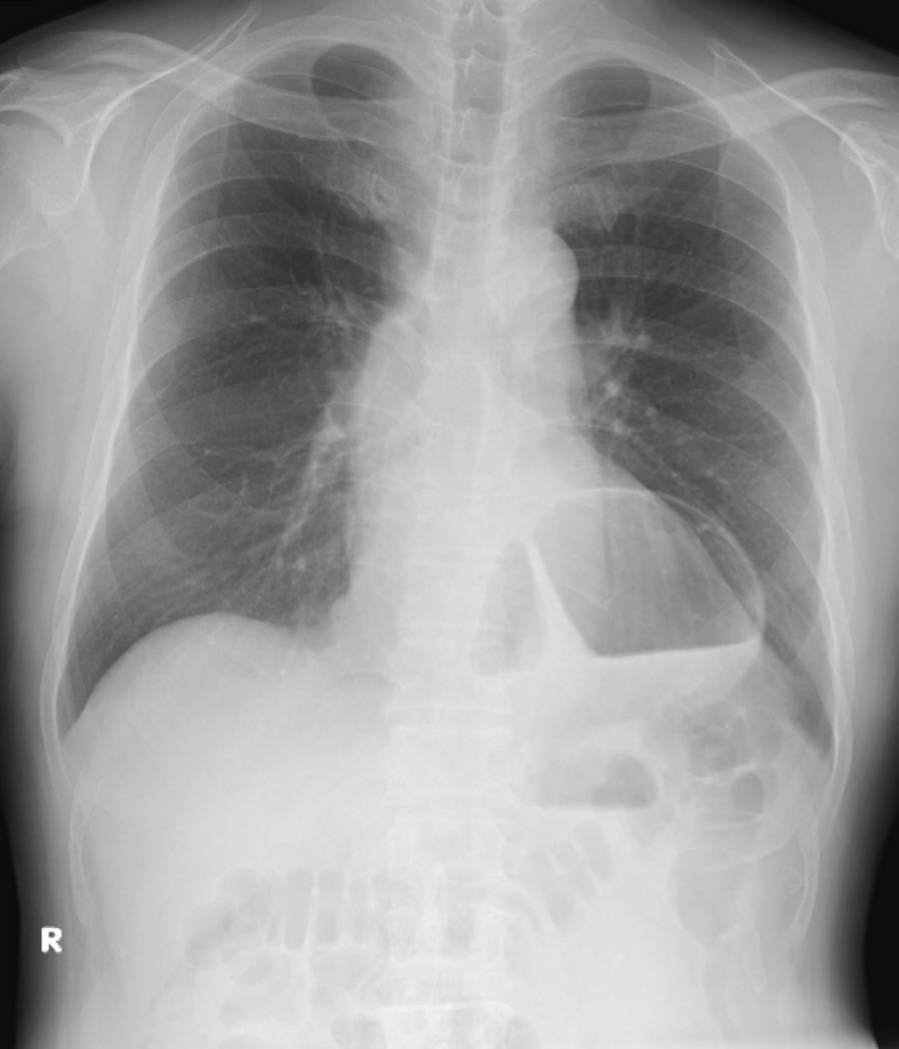
Plain chest radiograph on arrival. A plain chest radiograph at an upright position showed air above the left diaphragm overlapping with the cardiac shadow. This finding suggested a hiatal hernia

**Fig. 2:**
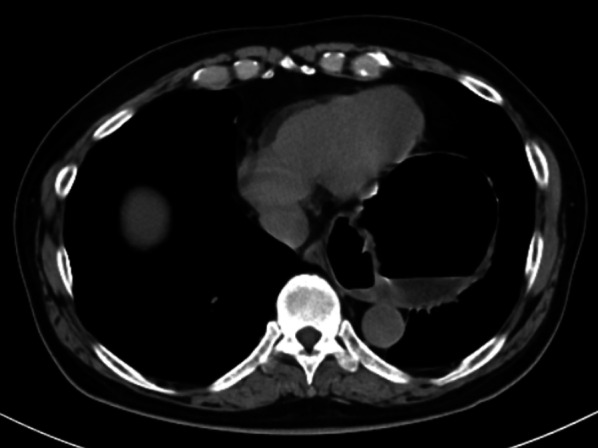
Plain computed tomography on arrival. Plain computed tomography showed that the body of the stomach presented a torsion and was sliding into the left thoracic cavity

**Fig. 3:**
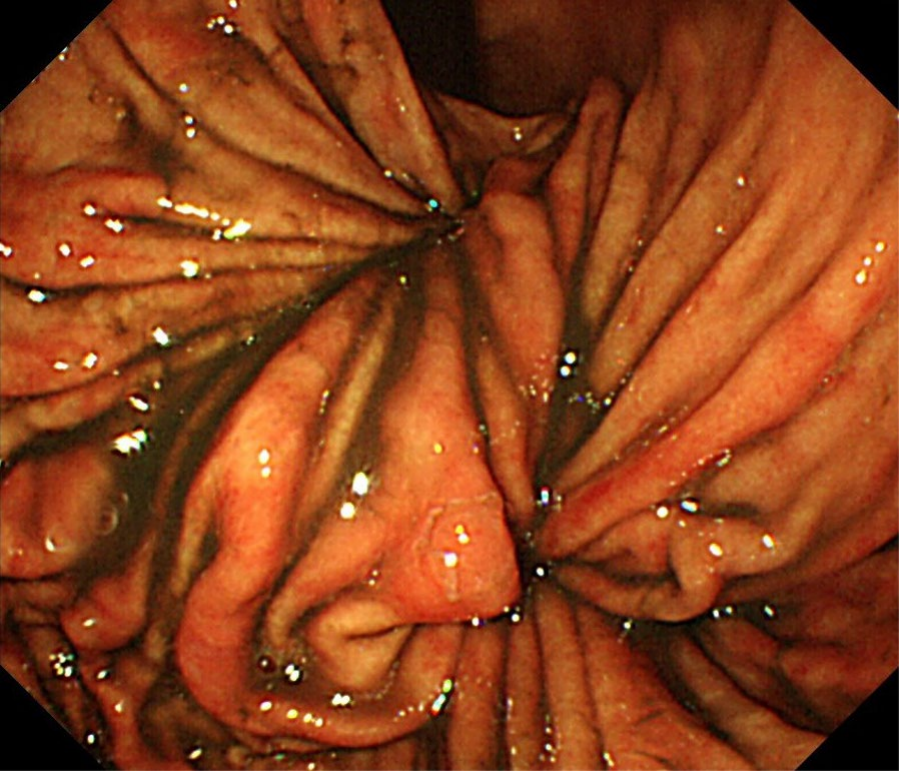
Esophagogastroduodenoscopy on arrival. The gastric volvulus was estimated to be mixed, about 90 degrees in the short axis and about 90 degrees in the long axis

His vital signs on the first visit were the following: body temperature, 36.6 ℃; blood pressure, 127/73 mmHg; and heart rate, 90 beats/min. Physical examination revealed tenderness in the epigastric area with a flat abdomen. Laboratory findings, which suggested mild inflammation and acute kidney injury (AKI) due to hypovolemia, were as follows: white blood cell count, 9470/mm^3^; aspartate aminotransferase, 45 U/L; alanine aminotransferase, 31 U/L; blood urea nitrogen, 76.7 mg/dL; creatinine, 1.87 mg/dL; amylase, 536 U/L; and C-reactive protein, 0.36 mg/dL. A chest radiograph showed air above the left diaphragm, just behind the cardiac shadow, and a hiatal hernia was suspected (Fig. 1). CT was performed without contrast enhancement because of the severe AKI and revealed no deviation or reduction of the mediastinal part of the esophagus, and the cardia was located in the abdominal cavity instead of sliding into the thoracic cavity. In contrast, the body of the stomach presented a torsion and was sliding toward the left thoracic cavity (Fig. 2). Although the chest radiography suggested a hiatal hernia, the CT findings revealed no characteristic signs of a sliding-type hiatal hernia. Moreover, only the body of the stomach was involved in the volvulus and slid into the left thoracic cavity. Therefore, we suspected a paraesophageal hiatal hernia or a diaphragmatic hernia. On the second day after admission, we performed an EGD to reduce the volvulus. The EG junction was almost normal. A whirl sign of a fold was present at the fundus of the stomach, suggesting a gastric volvulus. The gastric volvulus was estimated to be a mixed type, about 90° in both short and long axes (Fig. 3). We attempted to reduce the volvulus endoscopically but failed. No signs of ischemia or necrosis were identified in the stomach; however, the patient’s symptoms persisted. Laparoscopic surgery was performed on the third day after admission. We did not measure the esophageal pressure or monitor the pH because of the patient’s desire for early treatment.

**Fig. 4:**
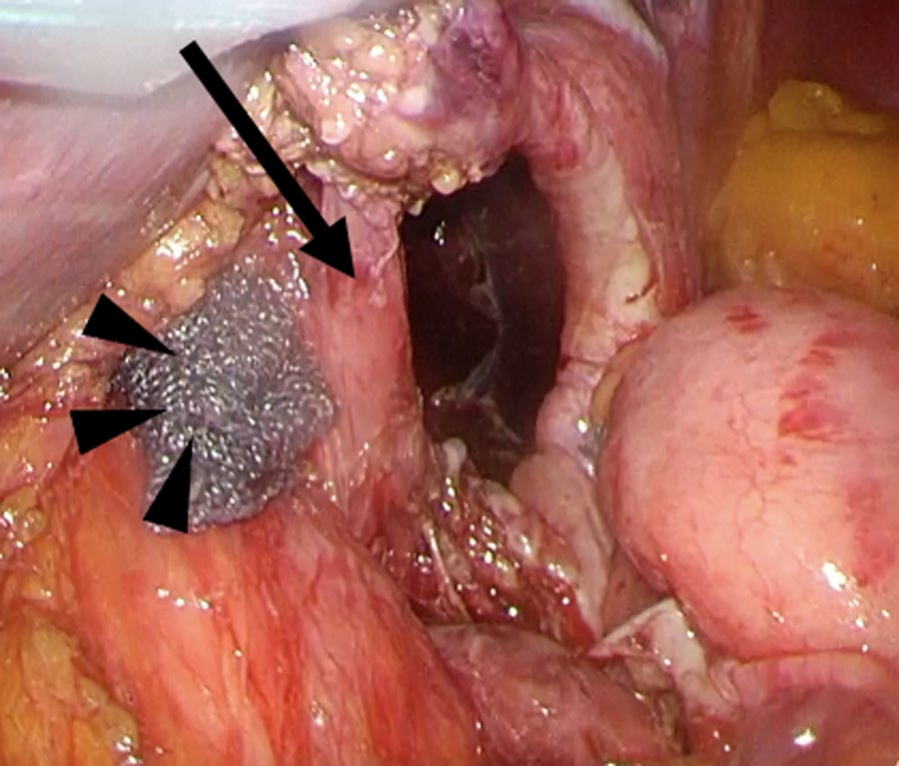
Hernial orifice and esophageal hiatus. We reduced the portion of the stomach that had deviated into the left thoracic cavity and were able to identify the left crus of the diaphragm (arrow) and esophageal hiatus (arrowheads). A hernial orifice was present at the left side of the left crus of the diaphragm

**Fig. 5:**
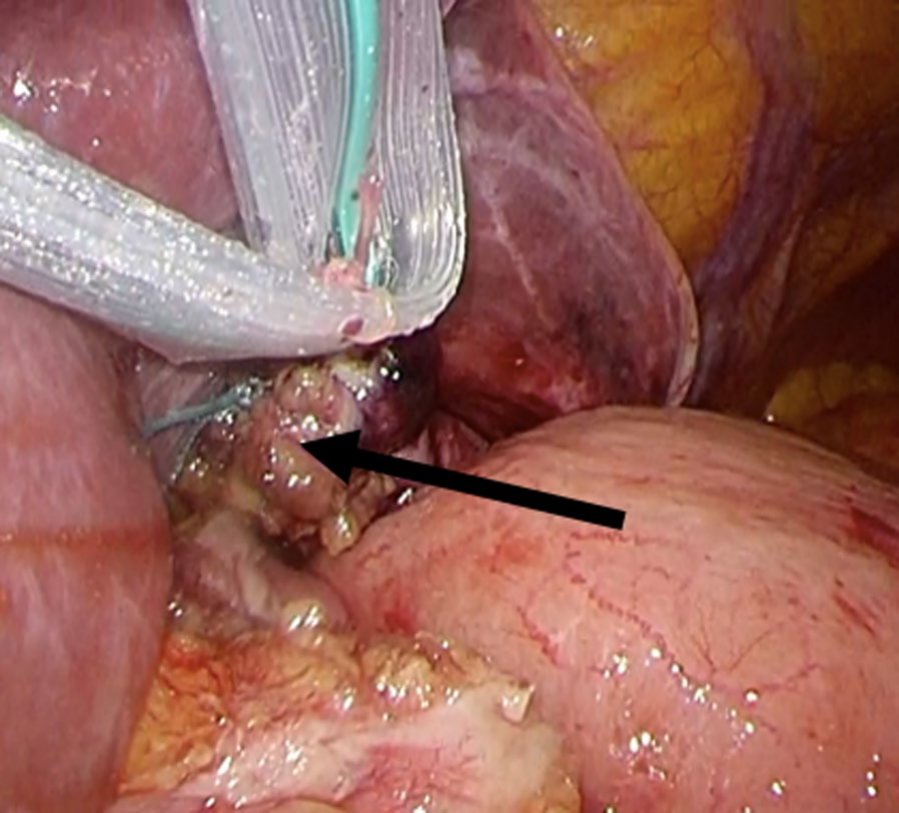
After repair of parahiatal hernia. We closed the orifice of the parahiatal hernia directly. We did not perform fundoplication, and only reconstructed the esophagogastric junction using sutures (arrow)

During the operation under general anesthesia, we used a trocar setting similar to that of usual laparoscopic gastrectomies (i.e., five-port setting). We reduced the prolapsed body of the stomach and detected a hernial orifice on the left side of the left crus of the diaphragm (Fig. 4). The stomach was not adherent to the hernial sac and the prolapsed portion was reduced easily. Therefore, a definite diagnosis of a parahiatal hernia, rather than paraesophageal, was made. The diameter of the hernial orifice was approximately 3 cm, and the hernial sac was thickened. Therefore, we closed the hernial orifice by direct simple closure using 2-0 non-absorbable threads. Adhesiolysis around the esophageal hiatus caused mild dilation of the angle of his and esophageal hiatus. Therefore, we sutured the esophageal hiatus using two 2-0 non-absorbable threads. The esophagus and fundus of the stomach were sutured to the crus of the diaphragm using three 3-0 absorbable threads. The EG junction was restored with these procedures (Fig. 5). The operative duration was 2 h 3 min and the bleeding volume was 10 mL.

**Fig. 6:**
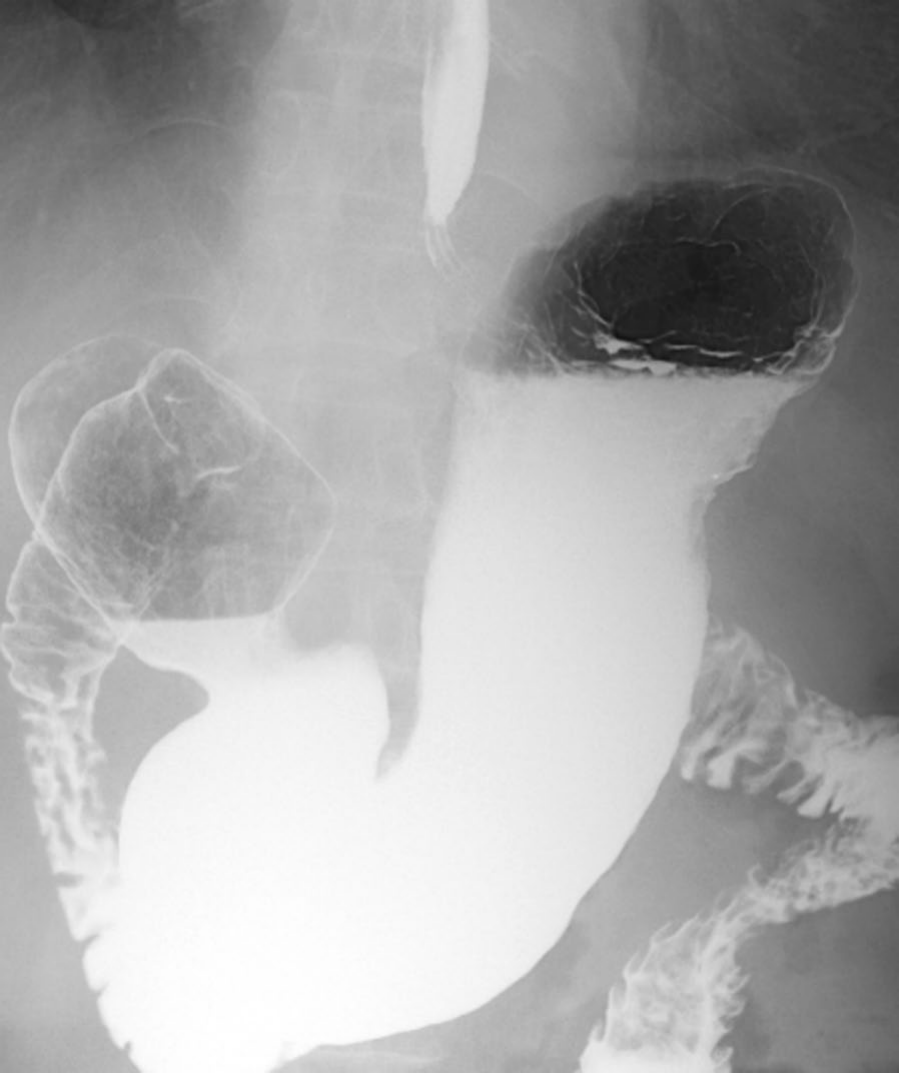
Esophago-gastrography after the surgery. In the second postoperative month, we performed an esophago-gastrography with contrast media, which did not reveal abnormal findings, including in the His angle

On postoperative day 5, we performed an esophago-gastrography with contrast media (Gastrografin®; BAYER, Osaka, Japan) and confirmed the absence of abnormal findings. Food intake after surgery was also high. The patient’s postoperative recovery was reasonable, and he was discharged on postoperative day 12. After being discharged from the hospital, the patient did not report any symptoms of gastroesophageal reflux disease (GERD). We evaluated his gastrointestinal system again approximately 2 months after the operation at our outpatient clinic and verified that the status was normal without disruption of the angle of his or gastroesophageal reflux (Fig. 6).

## Discussion

Diaphragmatic hernias, including parahiatal hernias, comprise both congenital and acquired types. The congenital have been reported to occur in 1 in 2500 newborns, with a survival rate of 67% [1]. Their most common pattern is one with the hernial orifice located only on the left diaphragm, followed by the right side only, and bilaterally [2]. Acquired diaphragmatic hernias are rare and account for approximately 5% of all traumatic injuries [1]. It is often diagnosed as symptomatic several years after injury. Parahiatal hernia is an extremely rare type of diaphragmatic hernia, in which the hernial orifice is formed adjacent to the esophageal hiatus. In cases with congenital etiology, it occurs due to the incompetent closure of a left pleuroperitoneal canal [3]; as a result, diaphragmatic tissue is present between the esophageal hiatus and the orifice of the hernia. The oldest case report employing the name “parahiatal hernia” dates back to 1962 [4]. Another case of a parahiatal hernia virtually described as a “Bochdalek hernia” has also been reported [5]. Such reports illustrate the confusion regarding the nomenclature of this disease.

**Fig. 7:**
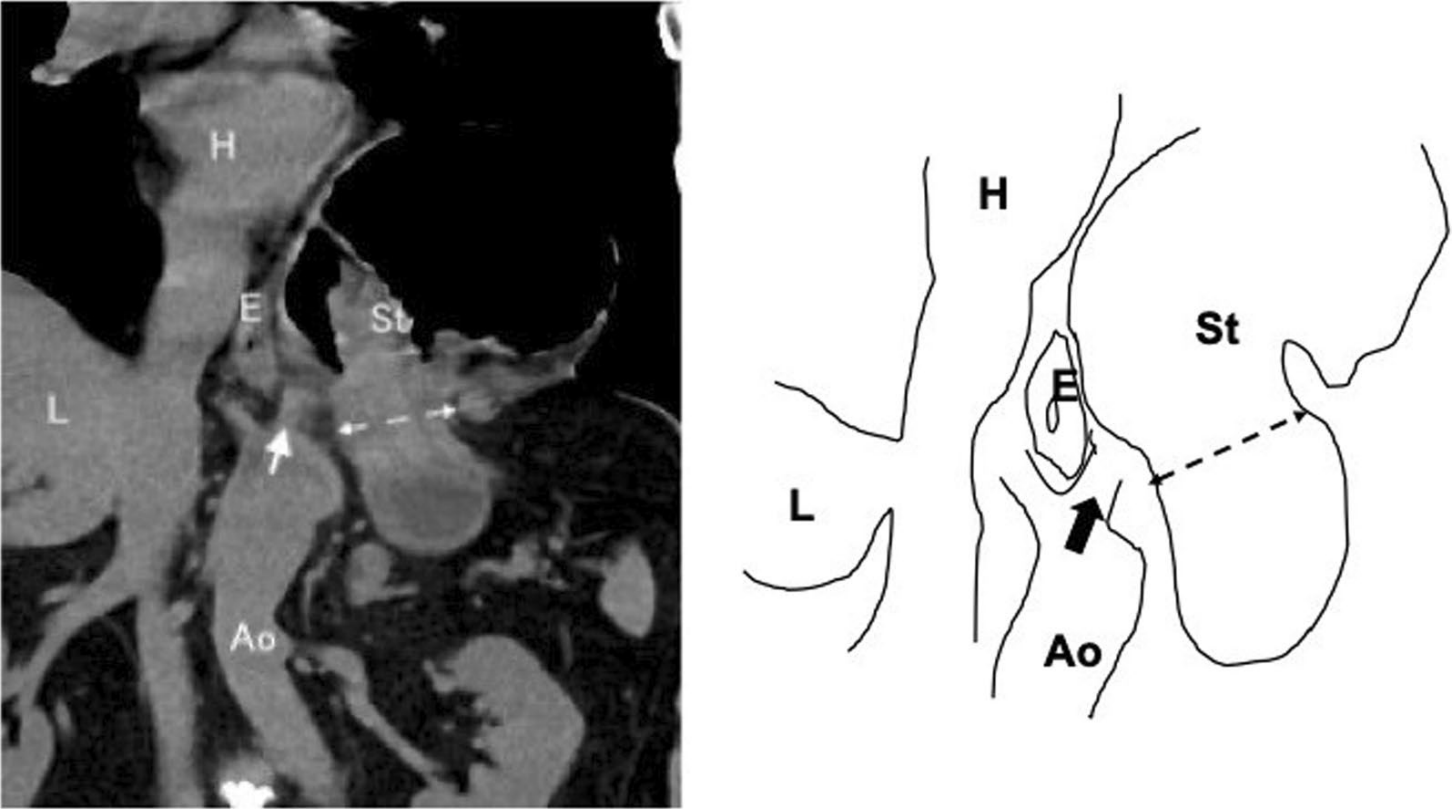
Reassuring plain computed tomography on admission. We retrospectively examined the details of preoperative plain computed tomography (coronal view) and were able to detect the body of the stomach deviated into the left thoracic cavity and the presence of connective tissue showing left diaphragmatic crus (arrow) beside the hernial orifice (double arrow dotted line) (H: heart, E: esophagus, L: liver, Ao: abdominal aorta, St: stomach)

On admission, we diagnosed this case as a special hiatal hernia that differed from other types of categorized hernias. This hernia was similar to a paraesophageal hiatal hernia, as the content of the hernia that prolapsed into the thoracic cavity was the body of the stomach, accompanied by a gastric volvulus. A hernial orifice was present on the left side of the esophageal hiatus, but not at the esophageal hiatus itself, according to intraoperative findings during laparoscopic surgery. Tissue was found in the left crus of the normal diaphragm between the orifice of the hernia and the esophageal hiatus. These intraoperative findings indicated a parahiatal hernia. In general, establishing a definitive preoperative diagnosis to plan the surgical details is essential. We considered the details of the disease preoperatively. Whether the hernial orifice can be directly closed, or whether fundoplication should be performed should be discussed. Recently, the definition of CT and magnetic resonance imaging (MRI) has improved, with various reconstructed images available. These technologies enable the assessment of the details of the surgical anatomy. In the present case, if we had identified the existence of tissue between the hernial orifice and the esophageal hiatus using preoperative imaging, a definite diagnosis could have been made. To date, few case reports of parahiatal hernias diagnosed preoperatively using CT or MRI, have been published [6, 7]. In our case, a definite diagnosis was established based on intraoperative findings. We then retrospectively evaluated the details of the preoperative images and detected the presence of soft tissue on the lateral side of the esophageal hiatus (Fig. 7).

The patient preferred definitive therapy promptly because of the severe epigastric pain he was experiencing. Therefore, we performed urgent laparoscopic exploration and did not conduct other complementary examinations. Laparoscopic surgery is less invasive than open surgery, enables the observation of details of the surgical anatomy, and improves the accuracy of the procedure owing to the magnified view of the operative field. In such aspects, laparoscopic surgery is better than open surgery, being especially effective in etiologies that require procedures in the deep parts of the trunk. Laparoscopic repair was selected in the most recent reports, and this approach is reasonable.

Here, we described the details of a surgery for parahiatal hernia. The first step was to reduce the body of the stomach, which had prolapsed from the abdominal cavity and return it to the thoracic cavity. The contents of the hernia rarely adhere to the sac, and reduction is easy. With regard to closing the hernial orifice, two possible methods are simple suturing and mesh closure. In cases with a small hernial orifice and no fragility in the surrounding tissue, simple closure using a non-absorbable thread is generally performed. We utilized 2-0 non-absorbable threads (ETHIBOND®) considering the visible findings. However, if the hernial orifice is large, a simple closure is insufficient, and mesh closure is considered. Some case reports have described laparoscopic repair of parahiatal hernias, and closure of the defect was performed using non-absorbable polypropylene, monofilament absorbable sutures, or a titanium hernia stapler [8, 9].

Doubts exist on whether fundoplication is necessary in patients with parahiatal hernias. Those with diaphragmatic hernias, including parahiatal hernias, do not present symptoms of GERD. This is different from patients with a sliding-type hiatal hernia [10, 11]. Therefore, fundoplication is generally not required in diaphragmatic hernia cases, including parahiatal hernias. In diaphragm embryogenesis, the mature diaphragm is derived from many embryonic elements, such as the septum transversum and pleuroperitoneal membrane [2]. In adults, an esophageal hernia can be associated with a parahiatal hernia, as reported by Muramatsu et al. [7]. In the presence of both hernias, fundoplication should be performed using the Toupet procedure. In our case, we performed adhesiolysis around the esophageal hiatus and EG junction to clarify the details of the herniation. Consequently, the EG junction was slightly disrupted. Therefore, we reconstructed the junction by reducing the size of the esophageal hiatus using sutures and suturing between the esophagus and cardia of the stomach. After being discharged from the hospital, the patient did not report any GERD symptoms. The site at which these procedures were performed was almost normal on postoperative radiographic guidance using esophagogastrography with contrast media. However, if we had established a definite preoperative diagnosis, we may not have performed excessive adhesiolysis around the esophagus and could have avoided reconstructing the EG junction.

**Fig. 8:**
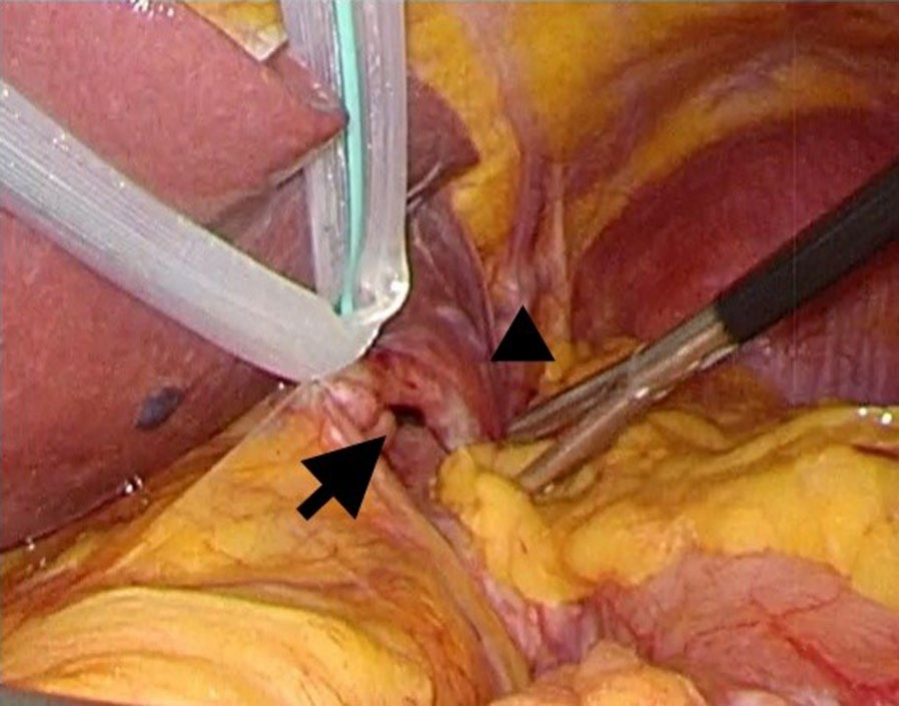
Transition area of left triangular ligament was located just above the hernial orifice (arrow), and the lateral segment of the liver (arrowhead) did not cover the left subdiaphragmatic space, including the hernial orifice

Finally, the patient noticed the symptoms of a hernia in his 60 s. He presented no apparent history of trauma, which was not sufficient to determine whether it was of congenital etiology. The reason for which patients with congenital diaphragmatic hernias develop symptoms in adulthood is unclear. A possible mechanism for the onset of a hernia may be due to age-related changes in body shape and organ size, which have not been discussed previously. Generally, elevated intra-abdominal pressure due to obesity or pregnancy, changes in the shape of the body from adolescence to middlescence, and exacerbation of the fragility of the hernial orifice cause the appearance of symptoms. In our case, the lateral area of the liver was relatively small based on intraoperative findings, and the surface of the diaphragm was widely exposed (Fig. 8). We hypothesized that the orifice of the parahiatal hernia was covered by the liver at a younger age and became exposed in the patient’s 60 s due to volume reduction of the liver. Although lean tissue of a liver decreases with aging [12], we were not able to find any reports of morphological atrophy of a liver. In terms of the hernial orifice, there is a possibility of atrophy of the left crus of the diaphragm. As this is simply a hypothesis from a single case, examining the pathogenesis of other cases and whether the topological or special changes in abdominal organs due to aging might be related to the onset of a parahiatal hernia, is of great interest. If it is congenital, why congenital conditions develop in the elderly is a subject for future study.

## Conclusions

Parahiatal hernias are very rare, and a definitive diagnosis remains difficult. Even in cases without a definite preoperative diagnosis, laparoscopic surgery might lead to an accurate diagnosis based on intraoperative findings. However, it is important to be aware of the existence of this rare disease to establish a definite preoperative diagnosis. Moreover, considering various types of imaging modalities, such as multi-detector-row CT, identifying the tissue of the diaphragm between the hernial orifice and the esophageal hiatus is feasible, and the disease can be treated safely and accurately.

## Data Availability

Not applicable.

## Abbreviations

EG junction: Esophagogastric junction
CT: Computed tomography
EGD: Esophagogastroduodenoscopy
AKI: Acute kidney injury
MRI: Magnetic resonance imaging
GERD: Gastroesophageal reflux disease

## References

[1] Testini M, Girardi A, Isernia RM, De Palma A, Catalano G, Pezzolla A, Gurrado A (2017). Emergency surgery due to diaphragmatic hernia: case series and review. World J Emerg Surg.

[2] Cannata G, Caporilli C, Grassi F, Perrone S, Esposito S (2021). Management of congenital diaphragmatic hernia (CDH): role of molecular genetics. Int J Mol Sci.

[3] MacDougall JT, Abbott AC, Goodhand TK (1963). Herniation through congenital diaphragmatic defects in adult. Can J Surg.

[4] Poppe JK, Rosenblatt MS (1962). Strangulated and perforated para hiatal hernia repaired with recovery. West J Surg Obstet Gynecol.

[5] Susmallian S, Raziel A (2017). A rare case of bochdalek hernia with concomitant para-esophageal hernia, repaired laparoscopically in an octogenarian. Am J Case Rep.

[6] Omori K, Miyata K, Yuasa N (2011). A case of parahiatal hernia diagnosed before operation. Jpn J Gastroenterol Surg.

[7] Muramatsu R, Nobuoka T, Ito T, Ogawa T, Korai T, Takemasa I (2022). Laparoscopic mesh repair and Toupet fundoplication for parahiatal hernia complicated by sliding hiatal hernia: a case report. Int J Surg Case Rep.

[8] Akiyama Y, Iwaya T, Endo F, Chiba T, Takahara T, Otsuka K, Nitta H, Koeda K, Mizuno M, Kimura Y, Sasaki A (2017). Laparoscopic repair of parahiatal hernia after esophagectomy: a case report. Surg Case Rep.

[9] Takemura M, Mayumi K, Ikebe T, Hamano G (2013). Laparoscopic repair of secondary parahiatal hernia with incarceration of the stomach: a case report. J Med Case Rep.

[10] Inaba K, Sakurai Y, Isogaki J, Komori Y, Uyama I (2011). Laparoscopic repair of hiatal hernia with mesenterioaxial volvulus of the stomach. World J Gastroenterol.

[11] Preda SD, Pătraşcu Ș, Ungureanu BS, Cristian D, Bințințan V, Nica CM, Calu V, Strâmbu V, Sapalidis K, Șurlin VM (2019). Primary parahiatal hernias: acase report and review of the literature. World J Clin Cases.

[12] Palmer AK, Jensen MD (2022). Metabolic changes in aging humans: current evidence and therapeutic strategies. J Clin Invest.

