# Correction: Laparoscopic repair of a primary parahiatal hernia combined with gastric volvulus: a case report and literature review

**DOI:** 10.1186/s40792-024-01952-4

**Published:** 2024-06-26

**Authors:** Hirotada Muramatsu, Hisashi Amaike, Rena Ogura, Kouichi Shirono, Noriyuki Kamiya

**Affiliations:** 1 Department of Surgery, Ito Municipal Hospital, 196-1 Oka, Ito, Shizuoka Japan; 2 Department of General Surgery, Tokyo Bay Urayasu Ichikawa Medical Center, 3-4-32 Toudaijima, Urayasu, Chiba Japan

**Correction: Surgical Case Reports (2024) 10:135** 10.1186/s40792-024-01931-9

Following publication of the original article [1], the authors reported an error in figures 7 and 8.

The original figure 7 was:
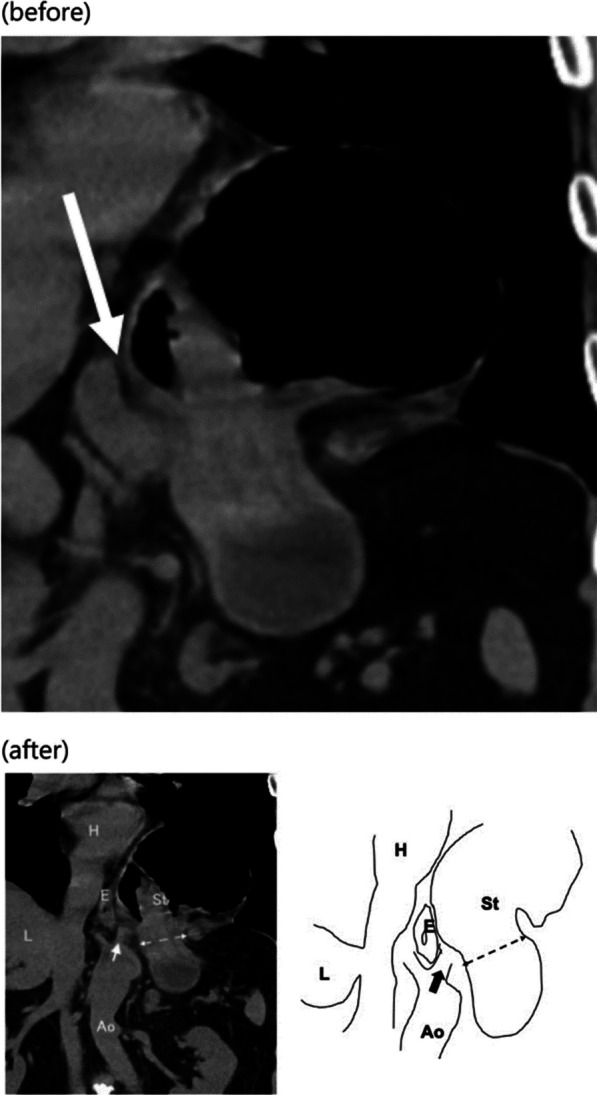


The correct Fig. 7 should read:

**Fig. 7:**
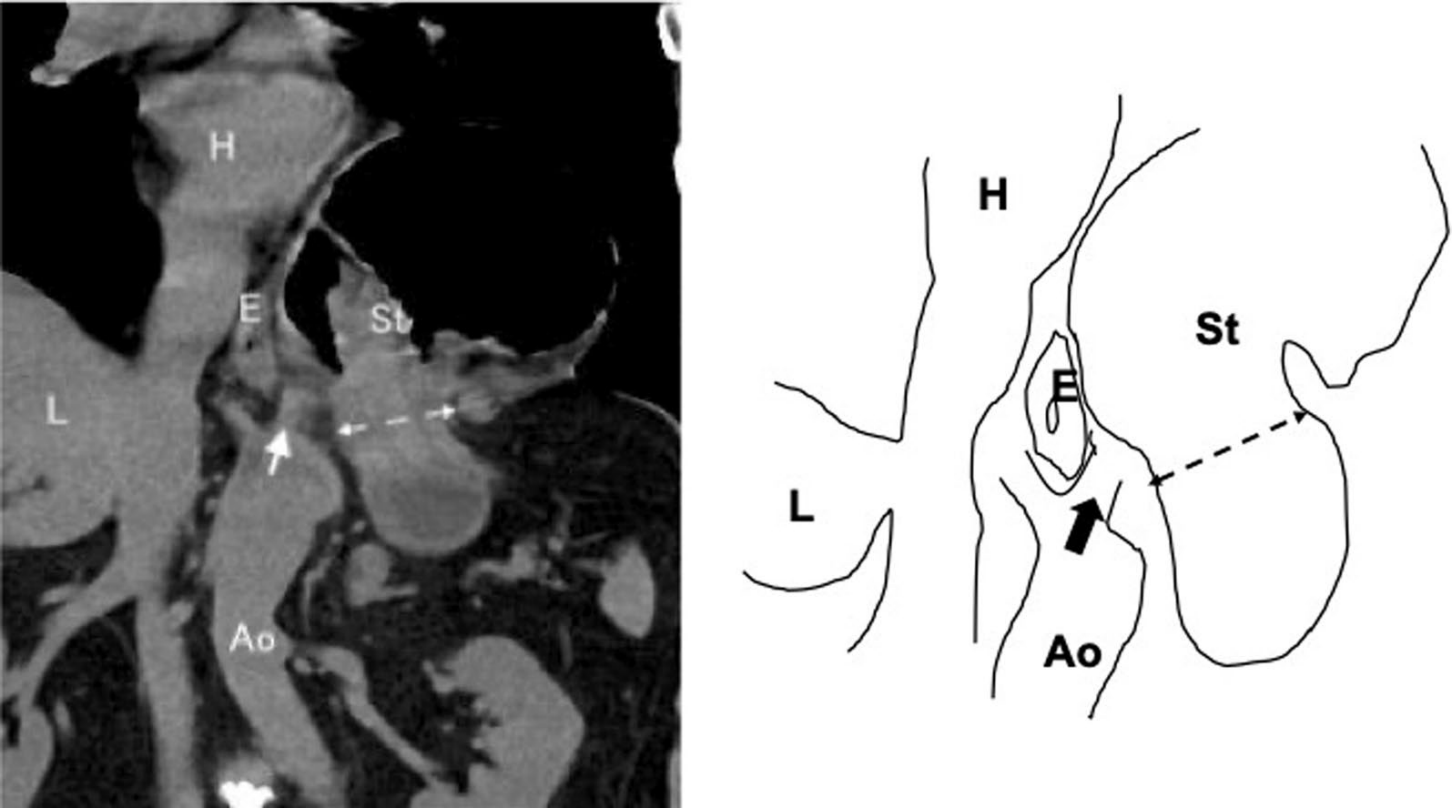
Reassuring plain computed tomography on admission. We retrospectively examined the details of preoperative plain computed tomography (coronal view) and were able to detect the body of the stomach deviated into the left thoracic cavity and the presence of connective tissue showing left diaphragmatic crus (arrow) beside the hernial orifice (double arrow dotted line) (H: heart, E: esophagus, L: liver, Ao: abdominal aorta, St: stomach)

The original figure 8 was:
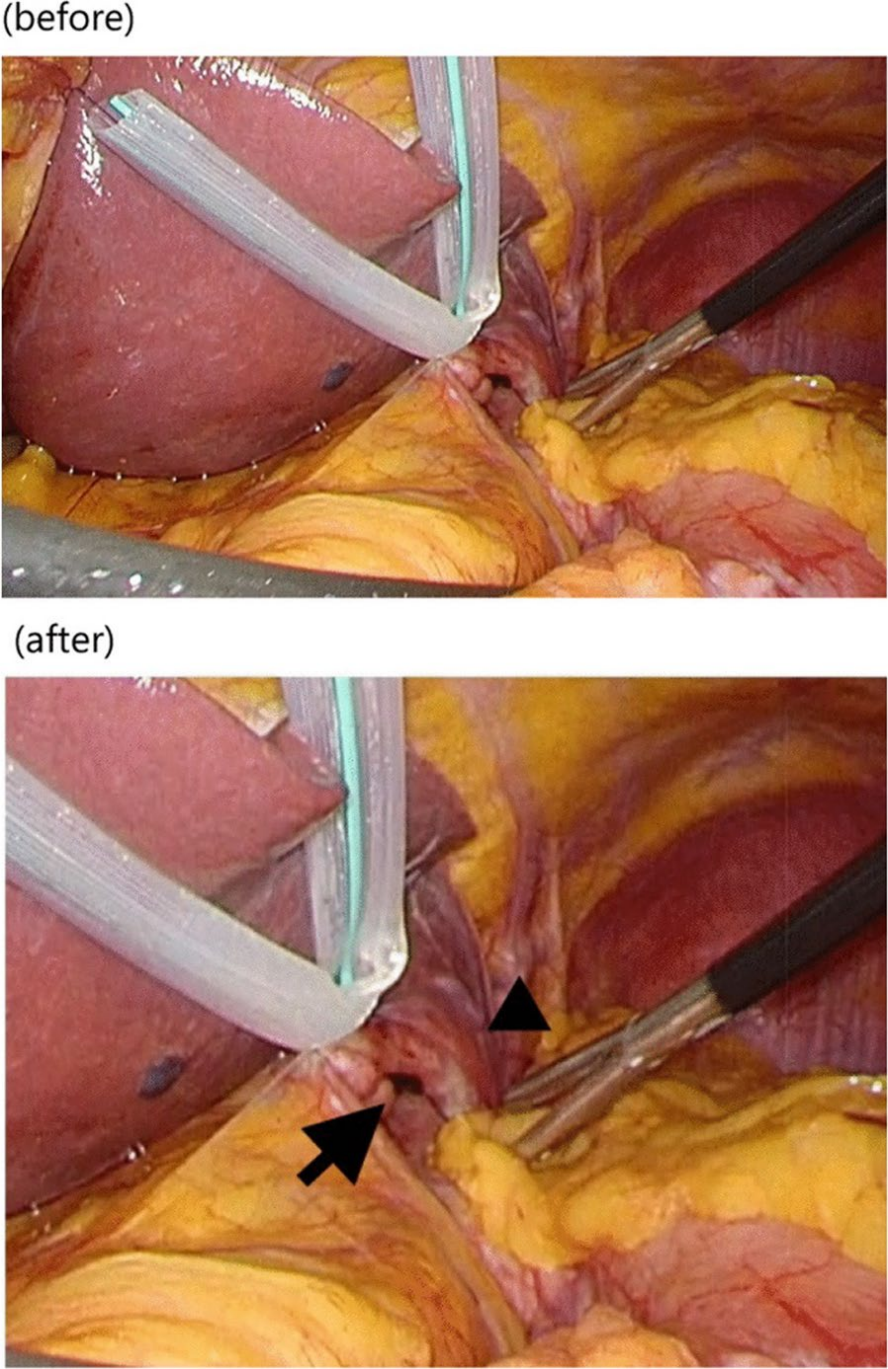


The correct Fig. 8 should read:

**Fig. 8:**
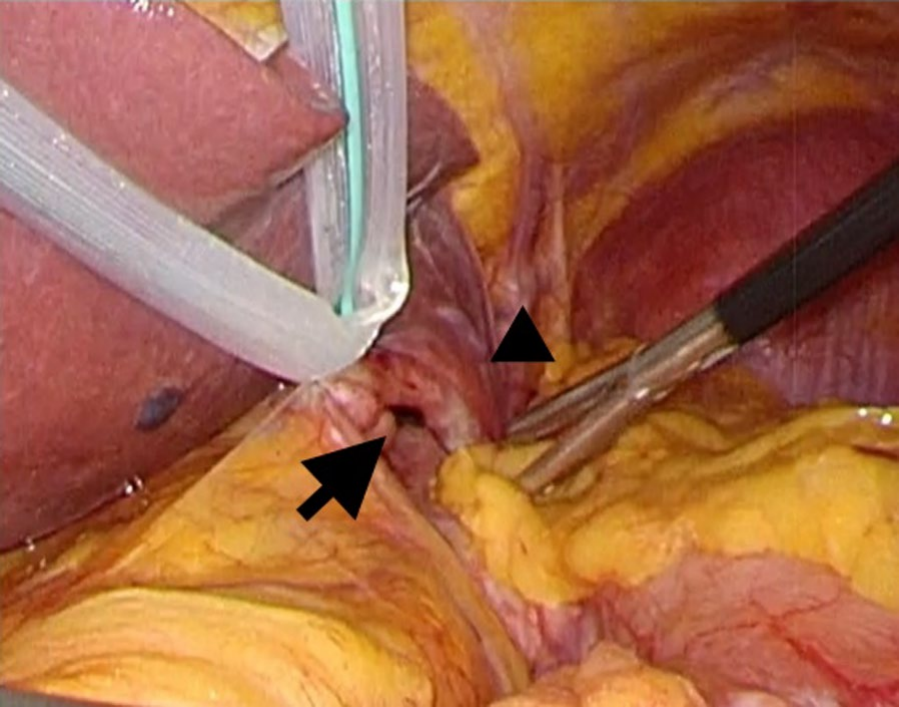
Transition area of left triangular ligament was located just above the hernial orifice (arrow), and the lateral segment of the liver (arrowhead) did not cover the left subdiaphragmatic space, including the hernial orifice

The original article [1] has been updated.
